# Canine Best disease as a translational model

**DOI:** 10.1038/s41433-024-03578-0

**Published:** 2025-01-07

**Authors:** Gustavo D. Aguirre, William A. Beltran

**Affiliations:** Division of Experimental Retinal Therapies, Department of Clinical Sciences, University of Pennsylvania, School of Veterinary Medicine, Philadelphia, PA 19104 USA

**Keywords:** Retinal diseases, Disease genetics

## Abstract

In this review, we summarize the findings of several pre-clinical studies in the canine *BEST1* disease model. To this end, client-owned and purpose bred dogs that were compound heterozygotes or homozygotes, respectively, for two or one of 3 different mutations in *BEST1* were evaluated by ophthalmic examination, cSLO/sdOCT imaging, and retinal immunohistochemistry to characterize the clinical and microanatomic features of the disease. Subsequently AAV-mediated gene therapy was done to transfer the *BEST1* transgene to the RPE under control of a *hVMD2* promoter. We demonstrated that canine bestrophinopathies are an RPE-photoreceptor interface disease with underdeveloped RPE apical microvilli that invest rod and cone outer segments. This leads to microdetachments which later progress to clinically evident RPE-retinal separation and a spectrum of disease stages, ranging from vitelliform to vitelliruptive/atrophic lesions, similar to Best Vitelliform Macular Dystrophy (BVMD). Gene therapy corrects the microdetachments and reverses large lesions when delivered at the pseudohypopyon stage of disease. Because of the similar clinical and microstructural abnormalities between the canine model and BVMD, and positive response to gene therapy, the canine model is a valuable translational model for developing gene and other therapies for BVMD.

## Introduction

Mutations in the Bestrophin 1 gene (*BEST1*, *VMD2*) cause several phenotypically distinct monogenic retinal disorders in man, the prototypical one being autosomal dominant Best Vitelliform Macular Dystrophy (BVMD) [1]. This form of Best disease has a characteristic macular lesion that progresses from a small elevated fried egg-like (vitelliform) lesion of the macula to a larger focal lesion that accumulates autofluorescent (AF) lipofuscin pigment, before undergoing the degenerative vitelliruptive and atrophic phases with loss of central acuity [2, 3].

While there is no question that mutations in *BEST1* cause retinal disorders, the specific function of the protein, and some of the pathways leading from mutant gene to disease, are not well understood. BEST1 is proposed as a Ca^++^-activated chloride channel [4], but this hypothesis has been challenged based on studies in *BEST1* knockout (KO) mice that found that Ca^++^-activated chloride channel currents are not abolished [5]. The problem with basing *BEST1*-mechanistic studies on mice is that the currently available KO mouse models fail to represent the essential features of the disease. These mice have no ocular phenotype, do not recapitulate the human disease, have no gross visual deficits or pathology, and have normal Cl^-^ currents in the RPE where the protein is expressed (see ref. [2] for review and ref. [5]).

A major limitation of the *BEST1* KO models is in translational applications, i.e. going from the cage to the bedside, using gene and other therapies. While it is possible to test therapeutic gene therapy vectors to establish specific expression in the target tissue, and stability of expression of the therapeutic transgene, it is not possible to determine if treatment prevents disease, or reverses disease once established, or, as well, how effective is long term correction of the disease following treatment as the KO mice have no retinal disease phenotype. Hence there is a need for a model that recapitulates the essential features of the patient disease. The canine bestrophinopathy models fill this void and their translational potential are summarized in the following sections.

## Subjects, results, discussion

Findings presented in this study come from examinations carried out in client-owned dogs seen at our clinic, and, primarily, from detailed clinical, structural and functional studies carried out in purpose bred research dogs maintained at the Retinal Disease Studies Facility of the University of Pennsylvania. Details of those studies, including the breeding, housing, and care of the dogs have been presented in several prior publications [6–10]. All protocols and studies have been approved by the University of Pennsylvania IACUC.

### Best1 disease models and clinical phenotypes

There are 3 Best disease canine models. Until the mutant gene and breed-specific mutations were identified, these autosomal recessive diseases were referred to as canine multifocal retinopathy (cmr1-cmr3) [11–13]. The first one, cmr1, represents a founder mutation in the English mastiff breed that is common in most large-sized dog breeds that originated from mastiffs [13]. The other two, cmr2 and 3, are ‘private’ mutations that are limited, respectively, to the Coton de Tulear and Lapponian herder breeds [12, 13]. Although these *Best1* mutations involve different domains of the protein {involving exons 2 (cmr1), 5 (cmr2) or 10 (cmr3)}, it is somewhat surprising that the resultant retinal phenotypes are similar (Table 1).

**Table 1:** Canine best disease mutations.

Disease variant	Mutation	Refences
Canine Best-cmr1	Exon 2/codon 25: C_73_T replaces arginine with stop.	[12]
Canine Best-cmr2	Exon 5: G_482_A results in Gly161Asp substitution.	[12]
Canine Best-cmr3	C_1388_del/Pro463fs and a linked G_1466_T/Gly489Val nucleotide substitution. The Gly489Val substitution leads to a stop codon within the Pro463fs altered reading frame.	[13]

**Fig. 1:**
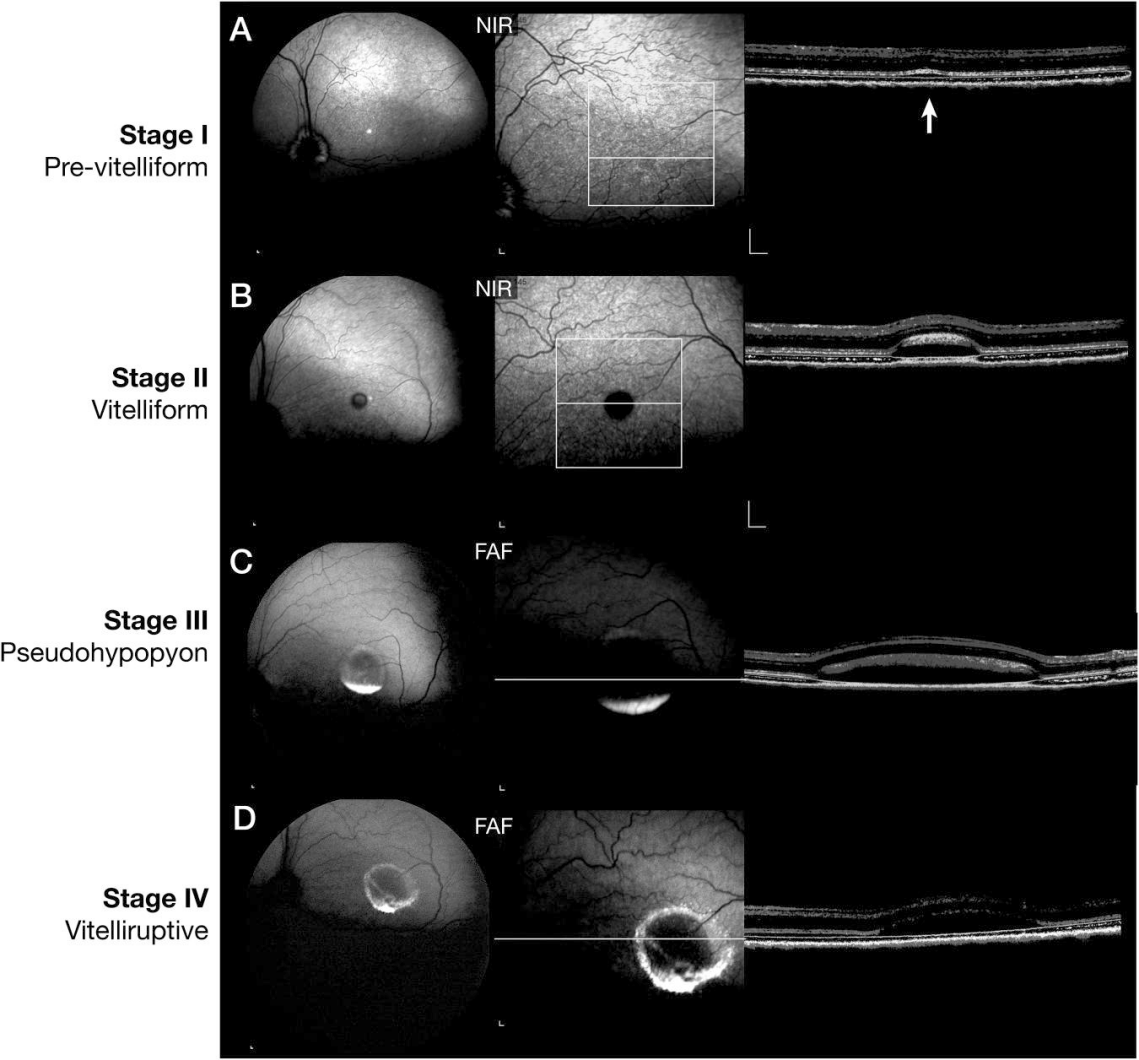
Clinical stages of canine *BEST1* disease. **A** Pre-vitelliform stage (Stage I). Near infrared (NIR) fundus image by cSLO (left), and single OCT b-scan showing a very small RPE-retinal separation (white arrow) not visible on fundus examination. **B** Vitelliform stage (Stage II). Near infrared (NIR) fundus image by cSLO (left), and single OCT b-scan showing a distinct RPE-retinal separation with debris accumulating over the photoreceptors. **C** Pseudohypopyon stage (Stage III). Fundus autofluorescence (FAF) with blue light image by cSLO shows expansion of the lesion and autofluorescent material settling to the inferior border of the large retinal lesion, and single OCT b-scan showing the debris material that is intermeshed with the photoreceptor layer. **D** Vitelliruptive stage (Stage IV). Fundus autofluorescence (FAF) with blue light image by cSLO shows that autofluorecent material is now redistributed and irregular, and single OCT b-scan shows a reduction of the debris material, and slight thinning of the ONL.

**Fig. 2:**
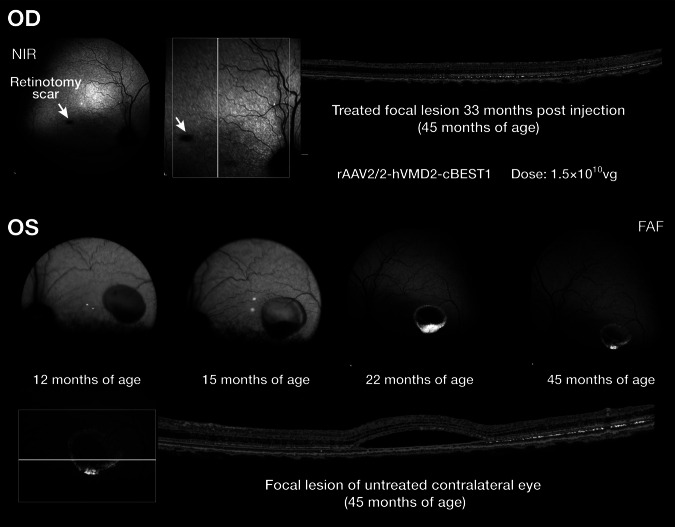
Treatment outcome following subretinal gene therapy with *AAV2/2-hVMD2-cBEST1.* OD. Eye treated at 12 months of age examined 33 months post injection. Near infrared (NIR) imaging shows a focal retinotomy scar (white arrow) and no evidence of the pseudohypopyon lesion present following treatment. OS. Fundus photographs (12 and 15 months of age), and fundus autofluorescence images (22 and 45 months) of the untreated fellow eye. By 45 months, of age the vitelliruptive stage has changed to atrophic stage (Stage V). At this stage, the outer nuclear layer has been markedly reduced in thickness in the fovea-like area and its immediate surrounding area centralis region.

Following the clinical staging classification for BVMD in man [3, 14], we have used similar phenotypic characteristics for staging the canine disease (Fig. 1). Retinal abnormalities are not found on routine ophthalmoscopic examination in the pre-vitelliform stage, although distinct focal separation, i.e. microdetachments, of the apical RPE from the photoreceptor outer segments is found by OCT (Fig. 1A). A distinct separation of the subretinal space is clearly evident in the vitelliform stage (Fig. 1B) which involves the canine fovea-like region [15] and extra foveal lesions [8, 12]. Blue autofluorescent (AF) material, presumably lipofuscin, accumulates in the pseudohypopyon stage (Fig. 1C), and this settles to the inferior border of the cystic appearing foveomacular lesion. In dogs, the fovea-like area and the immediately surrounding area centralis are analogous to the human foveomacular region. This AF material becomes more dispersed in the vitelliruptive stage with slight thinning of the outer nuclear layer (Fig. 1D). Marked thinning of the outer nuclear layer with disruption of photoreceptor layer and decreased AF material are present in the atrophic stage (Fig. 2, lower panel-OS).

### Characterization of subretinal lesion; fluid or gel matrix?

**Fig. 3:**
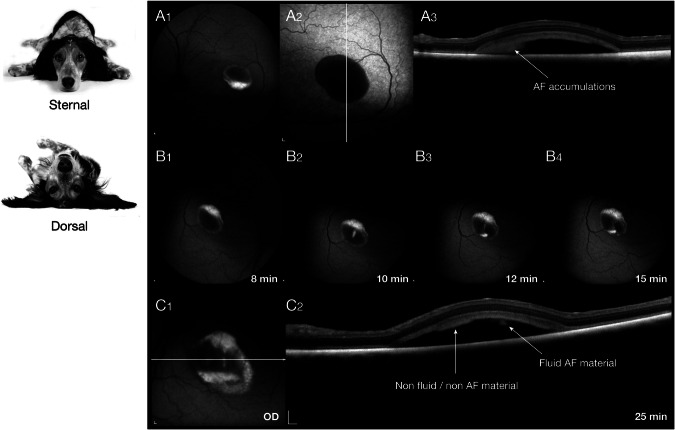
cSLO and sdOCT imaging done in the conventional imaging position (sternal) followed by rotating the dog to a dorsal position and repeating the scan. **A**_**1–3**_ Blue autofluorescent imaging (**A**_**1**_) shows autofluorescent material settled at the inferior border of the pseudohypopyon lesion. The single OCT b-scan shows that the debris material accumulates at the site of the autofluorescent material, and also covers the photoreceptor layer (**A**_**2-3**_). **B**_**1–4**_, **C**_**1, 2**_ When rotated to a dorsal scanning position, the autofluorescent material slowly redistributes through a funnel-shaped channel to the new inferior portion of the lesion. This process is slow and incomplete during the 25 min of scanning. This redistribution is observed in the single OCT b-scan image (C_2_).

While the early microdetachments appear to be modulated by light, ie. expanding in the light and contracting in the dark [8, 10], larger vitelliform and pseudohypopyon lesions remain unchanged. Expansion/contraction of lesions with light would suggest that the subretinal material in the small lesions is liquid and is readily transported into or out of the subretinal space. In contrast, it seems unlikely that the unchanged larger lesions have a fluid component. Because gene therapy would involve subretinal injections directed at the fovea-like region and surrounding area centralis, we felt important to characterize this material, at least clinically, as a means of informing therapeutic approaches and interpretation of outcome measures. To this end, we carried sdOCT/cSLO imaging with the dog placed in the conventional sternal recumbency position, followed by a repeat of the procedure in the same imaging session with the dog in dorsal recumbency. In the sternal position, autofluorescence imaging of a pseudohypyon lesion showed that the AF material accumulated in the inferior border of the cystic lesion (Fig. 3A_1_); OCT imaging shows the accumulated material mainly clustered in the lower quadrant, but also distributed throughout the subretinal space in close association with the photoreceptor layer (Fig. 3A_2, 3_). Sequential autofluorescence scans taken after the dog is placed in dorsal recumbency shows a very slow redistribution of the AF material (Fig. 3B_1–4_); it did not cascade down, as in a snow globe, but ‘trickled’ through a channel in a more solid gel or matrix. Even after 25 min in dorsal recumbency, the AF material is not fully distributed to the new inferior border of the lesion and continues to flow through the same channel (Fig. 3C_1, 2_).

### Structural abnormalities in the RPE-photoreceptor interface

**Fig. 4:**
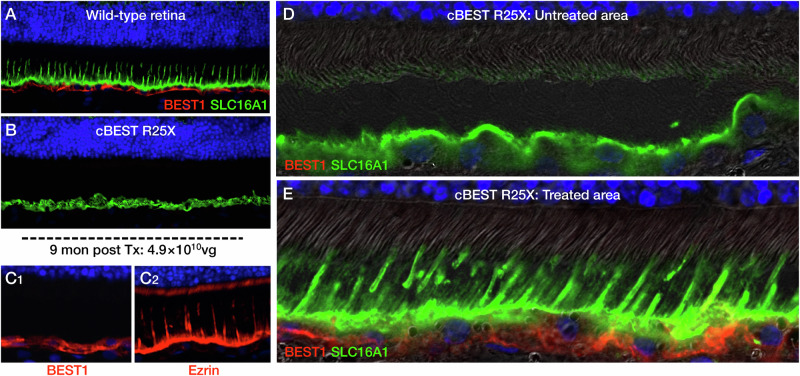
Abnormal RPE-photoreceptor interface in mutants is corrected by gene therapy. Immunohistochemistry (IHC) of the wild-type (**A**) and mutant (**B**, cmr1) RPE layer labeled with antibodies against BEST1 (red) and monocarboxylate transporter SLC16A1 (green). In the normal retina, BEST1 clearly is localized basally, and SLC16A1 is labeled throughout the RPE, and is especially prominent in the finger-like projections of the RPE-cone outer segment sheath. The mutant retina lacks BEST1 labeling, and the RPE appears to be disorganized and has no apical SLC16A1 labeled extensions. This is seen at higher magnification in an untreated area of a treated eye (**D**). **C**_**1,2**_ Following gene therapy, BEST1 is now expressed in the RPE cells, and Ezrin, another marker for RPE cells and the finger-like projections of the RPE-cone outer segment sheath, are clearly seen. **E**. Treated region of cmr1 mutant shows BEST1 expression and intense labeling of RPE cells and RPE-cone outer segment sheath with SLC16A1 antibody. These projections invest the cone outer segments for most of their length.

Canine bestrophinopathies represent an RPE-photoreceptor interface disease. The interphotoreceptor space is complex. It is bordered by 4 cellular elements which include the RPE apical microvilli, rod and cone inner/outer segments, and Müller cell fiber baskets. This space also contains soluble and insoluble components among which, respectively, include, IRBP and the insoluble rod and cone matrix [16, 17]. In mutant dogs, we find abnormal pan-retinal RPE-photoreceptor interface with an apparent loss of cone-ensheathing RPE apical processes and compromised cone-associated insoluble interphotoreceptor matrix (IPM) [9, 18]. The apical microvilli of rods are shorter, and the multilayered RPE-cone outer segment sheath [19] is abnormally short, and fails to envelop most of the cone outer segments. Studies in young affected dogs at the end of postnatal retinal differentiation showed that the RPE rod apical microvilli and the cone sheath failed to develop (ref. [18] and Fig. 4A, B). Furthermore, peanut agglutinin (PNA) labeling of the insoluble cone extracellular matrix sheath showed that the close association between the outer segment/RPE-cone sheath/PNA cone insoluble matrix was compromised as the intervening RPE cone sheath was absent [18]. In vitro studies of human induced PSC-RPE carried out in David Gamm’s laboratory showed that autosomal recessive bestrophinopathy cells had a paucity of rod microvilli, and those present were short [9]. As well, BVMD hiPSC-RPE cells examined by electron microscopy show a reduced number and marked shortening of the RPE apical microvilli. These studies support the strong phenotypic similarities between the canine and human diseases.

### BEST1 gene augmentation reverses disease and corrects structural abnormalities

Because of the success of AAV-mediated gene therapy for another primary RPE disease, *RPE65*-LCA [20], we examined if AAV-mediated *BEST1* gene augmentation would reverse the ongoing retinal abnormalities, and if expression/overexpression of the therapeutic transgene in the RPE was safe. Initially, we tested the AAV2/2 serotype used in *RPE65* therapy studies along with AAV2/1 serotype which in dogs was shown to effectively target RPE cells in the *RPE65* mutant dogs [21]. In this pilot study, AAV2/1-human VMD2-cBest which was co-injected with an AAV expressing GFPcaused severe and specific damage to cone cells in the treated area and was not considered further [22]. In contrast, subretinal administration of *AAV2/2-hVMD2-cBEST* to the wild type retina resulted in intense BEST1 expression in the RPE. Instead of localizing to the basolateral membranes of the RPE cells as the endogenous protein, the expressed transgene protein was also was located diffusely through the cytoplasm without any adverse effects to the RPE and neuroretina in a 4-6 week/6 month observation period [22]. Additionally, in the pilot study the *AAV2/2-hVMD2-cBEST* vector was tested in a heterozygote (cmr1^+/−^) dog without any adverse effects [22]. These pilot studies led to more formal studies examining the therapeutic efficacy of gene therapy for Best disease.

**Fig. 5:**
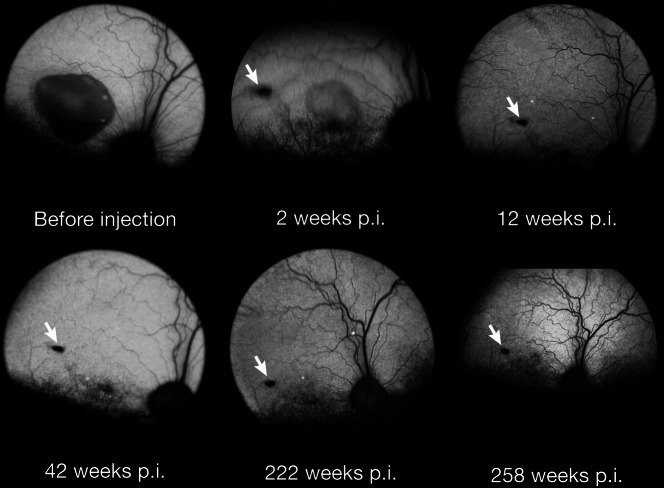
Fundus photographs showing a pseudohypopyon lesion in the *area centralis* of a dog at 1 year of age before injection, and at several time points following treatment. By 2 weeks post injection (p.i.), the retinal lesion is markedly decreased in size and elevation, and is flattened by 12 weeks p.i. The retina remains normal, and the retinotomy scar (white arrow) remains unchanged over an extended p.i. observation period.

For the formal proof of concept efficacy studies, subretinal injections of the *AAV2/2-hVMD2* vector with either the canine or human *BEST1* cDNA were carried out as previously detailed [8], and the injections were directed mainly to the fovea-like area and surrounding area centralis region. In all cases, the surgical bleb flattened within 24–48 hours post injection (p.i.). Untreated control eyes, or those injected with balanced salt solution (BSS) showed progression of the lesions. In contrast, mutant eyes treated with the therapeutic vector showed reversal of the lesions. Figure 5 shows one such dog injected at 12 months of age at the pseudohypopyon stage of disease. By 2 wks p.i, the large foveo-macular lesion decreased in size and the contents, present in the inferior border, also were diminished. Correction of the lesion was stable over several years as the retina in the treated area remained normal and attached with no evidence of any subretinal elevations or abnormalities. Only the focal retinotomy scar remained unchanged. In contrast, the retinal lesion in the untreated fellow eye continued to progress, and by 45 months of age the outer nuclear layer had thinned (atrophic stage) (Fig. 2). The development and progression of the fovea-like area and surrounding area centralis lesion over time in the untreated OS eye is illustrated in ref. [8], Fig. S4.

Following gene therapy, there is correction of the RPE-photoreceptor interface disease. There is expression of BEST1 protein in the RPE, both cytoplasmic and in basolateral membranes, and formation of new RPE cone outer segment sheaths which extend from the apical RPE to invest most of the cone outer segments (Fig. 4C_1_, C_2_ and E). Accompanying the correction the RPE-photoreceptor interface disease with gene therapy, the retinal microdetachments resolve [8].

Studies of canine BEST1 disease emphasize its value as a translational model. Even though the canine disease is autosomal recessive, and heterozygous dogs show no clinically evident phenotype, it is similar to human BVMD in terms of retinal phenotype and disease progression in the fovea-like area, and to autosomal recessive bestrophinopathy in extra foveal regions. The diseases in both species demonstrate many similarities in studies in which the same assessment methods are used. The dramatic and stable response to subretinal gene therapy, even in cases with large pseudohypopyon lesions, indicate that administering an injection to this area causes no damage, and lesion reversal occurs without adverse effects. It is remarkable that the retina in the foveo-macular region, which is already distended because of the lesion, has sufficient elasticity to accommodate a subretinal fluid injection without tearing. This is an important observation for planned Phase 1/2 clinical trial to be carried out in the near term. At present, the final nonclinical safety/efficacy studies are being completed in the BEST1 dog models.

## References

[1] Petrukhin K, Koisti MJ, Bakall B, Li W, Xie G, Marknell T, et al. Identification of the gene responsible for Best macular dystrophy. Nat Genet. 1998;19:241–7. 9662395 10.1038/915

[2] Hartzell HC, Qu Z, Yu K, Xiao Q, Chien LT. Molecular physiology of bestrophins: multifunctional membrane proteins linked to best disease and other retinopathies. Physiol Rev. 2008;88:639–72. 18391176 10.1152/physrev.00022.2007

[3] Johnson AA, Guziewicz KE, Lee CJ, Kalathur RC, Pulido JS, Marmorstein LY, et al. Bestrophin 1 and retinal disease. Prog Retin Eye Res. 2017;58:45–69. 28153808 10.1016/j.preteyeres.2017.01.006 PMC5600499

[4] Qu Z, Wei RW, Mann W, Hartzell HC. Two bestrophins cloned from Xenopus laevis oocytes express Ca(2+)-activated Cl(-) currents. J Biol Chem. 2003;278:49563–72. 12939260 10.1074/jbc.M308414200

[5] Marmorstein LY, Wu J, McLaughlin P, Yocom J, Karl MO, Neussert R, et al. The light peak of the electroretinogram is dependent on voltage-gated calcium channels and antagonized by bestrophin (best-1). J Gen Physiol. 2006;127:577–89. 16636205 10.1085/jgp.200509473 PMC2151522

[6] Guziewicz KE, Slavik J, Lindauer SJ, Aguirre GD, Zangerl B. Molecular Consequences of BEST1 Gene Mutations in Canine Multifocal Retinopathy Predict Functional Implications for Human Bestrophinopathies. Invest Ophthalmol Vis Sci. 2011;52:4497–505. 21498618 10.1167/iovs.10-6385 PMC3175949

[7] Singh R, Kuai D, Guziewicz KE, Meyer J, Wilson M, Lu J, et al. Pharmacological Modulation of Photoreceptor Outer Segment Degradation in a Human iPS Cell Model of Inherited Macular Degeneration. Mol Ther. 2015;23:1700–11. 26300224 10.1038/mt.2015.141 PMC4817951

[8] Guziewicz KE, Cideciyan AV, Beltran WA, Komaromy AM, Dufour VL, Swider M, et al. BEST1 gene therapy corrects a diffuse retina-wide microdetachment modulated by light exposure. Proc Natl Acad Sci USA. 2018;115:E2839–48. 29507198 10.1073/pnas.1720662115 PMC5866594

[9] Guziewicz KE, Sinha D, Gomez NM, Zorych K, Dutrow EV, Dhingra A, et al. Bestrophinopathy: An RPE-photoreceptor interface disease. Prog Retin Eye Res. 2017;58:70–88. 28111324 10.1016/j.preteyeres.2017.01.005 PMC5441932

[10] Wu V, Swider M, Sumaroka A, Dufour VL, Vance JE, Aleman TS, et al. Retinal response to light exposure in BEST1-mutant dogs evaluated with ultra-high resolution OCT. Vis Res. 2024;218:108379. 38460402 10.1016/j.visres.2024.108379 PMC11009038

[11] Grahn BH, Philibert H, Cullen CL, Houston DM, Semple HA, Schmutz SM. Multifocal retinopathy of Great Pyrenees dogs. Vet Ophthalmol. 1998;1:211–21. 11397233 10.1046/j.1463-5224.1998.00041.x

[12] Guziewicz KE, Zangerl B, Lindauer SJ, Mullins RF, Sandmeyer LS, Grahn BH, et al. Bestrophin gene mutations cause canine multifocal retinopathy: a novel animal model for best disease. Invest Ophthalmol Vis Sci. 2007;48:1959–67. 17460247 10.1167/iovs.06-1374 PMC1931491

[13] Zangerl B, Wickstrom K, Slavik J, Lindauer SJ, Ahonen S, Schelling C, et al. Assessment of canine BEST1 variations identifies new mutations and establishes an independent bestrophinopathy model (cmr3). Mol Vis. 2010;16:2791–804. 21197113 PMC3008713

[14] Boon CJ, Klevering BJ, Leroy BP, Hoyng CB, Keunen JE, den Hollander AI. The spectrum of ocular phenotypes caused by mutations in the BEST1 gene. Prog Retin Eye Res. 2009;28:187–205. 19375515 10.1016/j.preteyeres.2009.04.002

[15] Beltran WA, Cideciyan AV, Guziewicz KE, Iwabe S, Swider M, Scott EM, et al. Canine retina has a primate fovea-like bouquet of cone photoreceptors which is affected by inherited macular degenerations. PLoS One. 2014;9:e90390. 24599007 10.1371/journal.pone.0090390 PMC3944008

[16] Ishikawa M, Sawada Y, Yoshitomi T. Structure and function of the interphotoreceptor matrix surrounding retinal photoreceptor cells. Exp Eye Res. 2015;133:3–18. 25819450 10.1016/j.exer.2015.02.017

[17] Mieziewska KE, van Veen T, Murray JM, Aguirre GD. Rod and cone specific domains in the interphotoreceptor matrix. J Comp Neurol. 1991;308:371–80. 1865006 10.1002/cne.903080305

[18] Guziewicz KE, McTish E, Dufour VL, Zorych K, Dhingra A, Boesze-Battaglia K, et al. Underdeveloped RPE Apical Domain Underlies Lesion Formation in Canine Bestrophinopathies. Adv Exp Med Biol. 2018;1074:309–15. 29721958 10.1007/978-3-319-75402-4_38 PMC6035728

[19] Steinberg R, Wood I. Pigment Epithelial Cell Ensheathment of Cone Outer Segments in the Retina of the Domestic Cat. Proc R Soc Lond B. 1974;187:461–78. 4155505 10.1098/rspb.1974.0088

[20] Acland GM, Aguirre GD, Ray J, Zhang Q, Aleman TS, Cideciyan AV, et al. Gene therapy restores vision in a canine model of childhood blindness. Nat Genet. 2001;28:92–5. 11326284 10.1038/ng0501-92

[21] Acland GM, Aguirre GD, Bennett J, Aleman TS, Cideciyan AV, Bennicelli J, et al. Long-term restoration of rod and cone vision by single dose rAAV-mediated gene transfer to the retina in a canine model of childhood blindness. Mol Ther. 2005;12:1072–82. 16226919 10.1016/j.ymthe.2005.08.008 PMC3647373

[22] Guziewicz KE, Zangerl B, Komaromy AM, Iwabe S, Chiodo VA, Boye SL, et al. Recombinant AAV-mediated BEST1 transfer to the retinal pigment epithelium: analysis of serotype-dependent retinal effects. PLoS One. 2013;8:e75666. 24143172 10.1371/journal.pone.0075666 PMC3797066

